# Comparison of peripapillary retinal nerve fiber layer and macular thickness in non-diabetic chronic kidney disease and controls

**DOI:** 10.1371/journal.pone.0266607

**Published:** 2022-04-06

**Authors:** Jun Yong Chow, Poh Fong She, Xu Kent Pee, Wan Norliza Wan Muda, Mae-Lynn Catherine Bastion

**Affiliations:** 1 Faculty of Medicine, Department of Ophthalmology, Universiti Kebangsaan Malaysia Medical Center, Cheras, Kuala Lumpur, Malaysia; 2 Department of Ophthalmology, Hospital Tengku Ampuan Afzan, Ministry of Health, Kuantan, Pahang, Malaysia; 3 Department of Ophthalmology, Hospital Umum Sarawak, Ministry of Health, Kuching, Sarawak, Malaysia; University of Houston, College of Optometry, UNITED STATES

## Abstract

**Objective:**

This study aimed to compare the peripapillary retinal nerve fiber layer (pRNFL) thickness and macular thickness (MT) between patients with non-diabetic chronic kidney disease (NDCKD) and controls, as well as between different stages of NDCKD. We also evaluated the correlation between pRNFL thickness and MT with duration of NDCKD.

**Methods:**

This was a comparative cross-sectional study. Subjects were divided into NDCKD and control groups. Both pRNFL thickness and MT, including center subfield thickness (CST), average MT as well as average ganglion cell-inner plexiform layer (GC-IPL) were measured using spectral-domain optical coherence tomography. One-way ANCOVA test was used to compare the differences in pRNFL and MT between NDCKD and controls, as well as between the different stages of NDCKD. Spearman rank-order correlation coefficients were employed to determine the effects of NDCKD duration on pRNFL thickness and MT.

**Results:**

A total of 132 subjects were recruited, 66 with NDCKD and 66 controls. There was a statistically significant difference in superior (110.74 ± 23.35 vs 117.36 ± 16.17 μm, p = 0.022), nasal (65.97 ± 12.90 vs 69.35 ± 10.17 μm, p = 0.006), inferior quadrant (117.44 ± 23.98 vs 126.15 ± 14.75 μm, p = 0.006), average pRNFL (90.36 ± 14.93 vs 95.42 ± 9.87 μm, p = 0.005), CST (231.89 ± 26.72 vs 243.30 ± 21.05 μm, p = 0.006), average MT (268.88 ± 20.21 vs 274.92 ± 12.79 μm, p = 0.020) and average GC-IPL (75.48 ± 12.44 vs 81.56 ± 6.48, p = 0.001) values between the NDCKD group and controls. The superior quadrant (p = 0.007), nasal quadrant (p = 0.030), inferior quadrant (p = 0.047), average pRNFL (p = 0.006), average MT (p = 0.001) and average GC-IPL (p = 0.001) differed significantly between different stages of NDCKD. There was no correlation between pRNFL thickness and MT with duration of NDCKD.

**Conclusion:**

CST, average MT, average GC-IPL thickness, average pRNFL and all quadrants of pRNFL except the temporal quadrant were significantly thinner in NDCKD patients compared to controls. These changes were associated with the severity of CKD, but not its duration.

## Introduction

Chronic kidney disease (CKD) is rapidly emerging as a global public health problem, with a global prevalence rate of 9.1% [1]. This rate marks an increase of almost 30% over the past three decades, reflecting the demographics of an ageing population worldwide [2]. The morbidity of CKD stems not only from the decreased productivity and increased costs associated with the need for regular dialysis, particularly in resource-limited settings, but also from the increase in cardiovascular disease-related mortality among these patients [3].

CKD is defined as an estimated glomerular filtration rate (eGFR) of less than 60 ml/min/1.73 m2 present for more than three months, with or without evidence of kidney damage [4]. Although renal microvascular changes can only be assessed reliably via renal biopsy, the retina shares many features in common with the kidney, and the status of the retinal microcirculation is often used as a proxy for that of the renal vasculature [5–7]. CKD has been associated with narrowing of the retinal vessel calibers [7, 8]. A reduction in retinal vessel calibers is associated with reduced peripapillary retinal nerve fiber layer (pRNFL) thickness, as evaluated non-invasively via optical coherence tomography (OCT) [9, 10]. This may confound ophthalmological evaluation among patients with CKD [11], who may experience a spectrum of eye complaints including tear film instability, calcium deposits in the conjunctiva and cornea, cataract, retinopathy, glaucoma, and optic neuropathy [12–17].

Unfortunately, data on pRNFL and macular thickness (MT) changes in non-diabetic CKD (NDCKD) remains scarce. This study thus aimed to compare the pRNFL and MT between patients with NDCKD and controls, as well as between different stages of NDCKD. We also aimed to evaluate the correlation of pRNFL and MT with the duration of NDCKD.

## Materials and methods

### Study design

This was a comparative, cross-sectional observational study. The study period was from January 2020 to July 2021. Subjects were recruited using convenience sampling from the nephrology clinic, medical clinic, hemodialysis center, ophthalmology clinic and hospital staff. Ethical approval was obtained from the Research and Ethics Committee, Faculty of Medicine, Universiti Kebangsaan Malaysia (JEP-2020-069) and the Medical Research and Ethics Committee (MREC), Ministry of Health Malaysia (NMRR-19-3630-52263). The study was conducted in accordance with the tenets of the Declaration of Helsinki and the Malaysian Guidelines for Good Clinical Practice (GCP). Written informed consent was obtained from all subjects.

### Participants

The inclusion criteria for both NDCKD and control groups was an age between 18 to 65 years and subjects with underlying hypertension. All subjects underwent calculation of their eGFR based on the 2009 CKD-epidemiology (CKD-EPI) creatinine equation [18]. The Kidney Disease Improving Global Outcomes (KDIGO) 2012 guideline described 5 stages of eGFR categories, measured in ml/min/1.73 m^2^; G1 (eGFR ≥90); G2 (eGFR 60–89); G3a (eGFR 45–59); G3b (eGFR 30–44); G4 (eGFR 15–29) and G5 (eGFR < 15), also known as end-stage renal failure [19]. Those with an eGFR of less than 60 ml/min/1.73 m^2^ were included in the NDCKD group. The NDCKD group were further subdivided into three categories based on their latest eGFR (G3, G4 and G5). The control group was hypertensive patients without CKD.

We excluded patients with diabetes mellitus, neurodegenerative disease, bronchial asthma, chronic obstructive lung disease and obstructive sleep apnea, and intracranial pathology. Other exclusion criteria included high refractive error (±5.0 diopters spherical or ±2 diopters cylinder), optic disc abnormalities, optic neuropathy, glaucoma, ocular hypertension (intraocular pressure above 21 mmHg), glaucoma suspect (vertical and horizontal cup-to-disc ratio above 0.6, or asymmetry above 0.2 between the eyes), media opacity leading to poor signal strength (less than seven in OCT), inability to fixate, orbital disease, previous posterior segment surgery and any retinal disease such as diabetic retinopathy, retinal vein occlusion, age-related macular degeneration, macula hole, and epiretinal membrane.

### Blood pressure measurement

Blood pressure was measured using an automated digital sphygmomanometer (Collin Press-mate BP-8800, Colin Corporation, Japan) with an appropriate-sized cuff after 5 minutes of rest. The average systolic and diastolic blood pressure was based on two readings. A third reading was obtained if the discrepancy in systolic blood pressure between the first two readings was greater than 10 mm Hg or if the difference in diastolic blood pressure between the readings was more than 5 mm Hg.

### Ocular examination and OCT measurements

A comprehensive ophthalmologic examination including monocular distance visual acuity using a Snellen chart (Reichert; NY) at six meters, refraction, slit-lamp biomicroscopic examination, measurement of intraocular pressure using a Goldmann tonometer and dilated funduscopic examination was performed on the same day of recruitment. The examination and OCT measurements were done on a non-dialysis day or prior to hemodialysis (HD) to avoid any parameter changes due to the effects of HD [20, 21]. pRNFL thickness and MT were measured using Spectral Domain Cirrus OCT Model 4000 (Carl Zeiss Meditech, InC., Dublin, USA).

**Fig 1:**
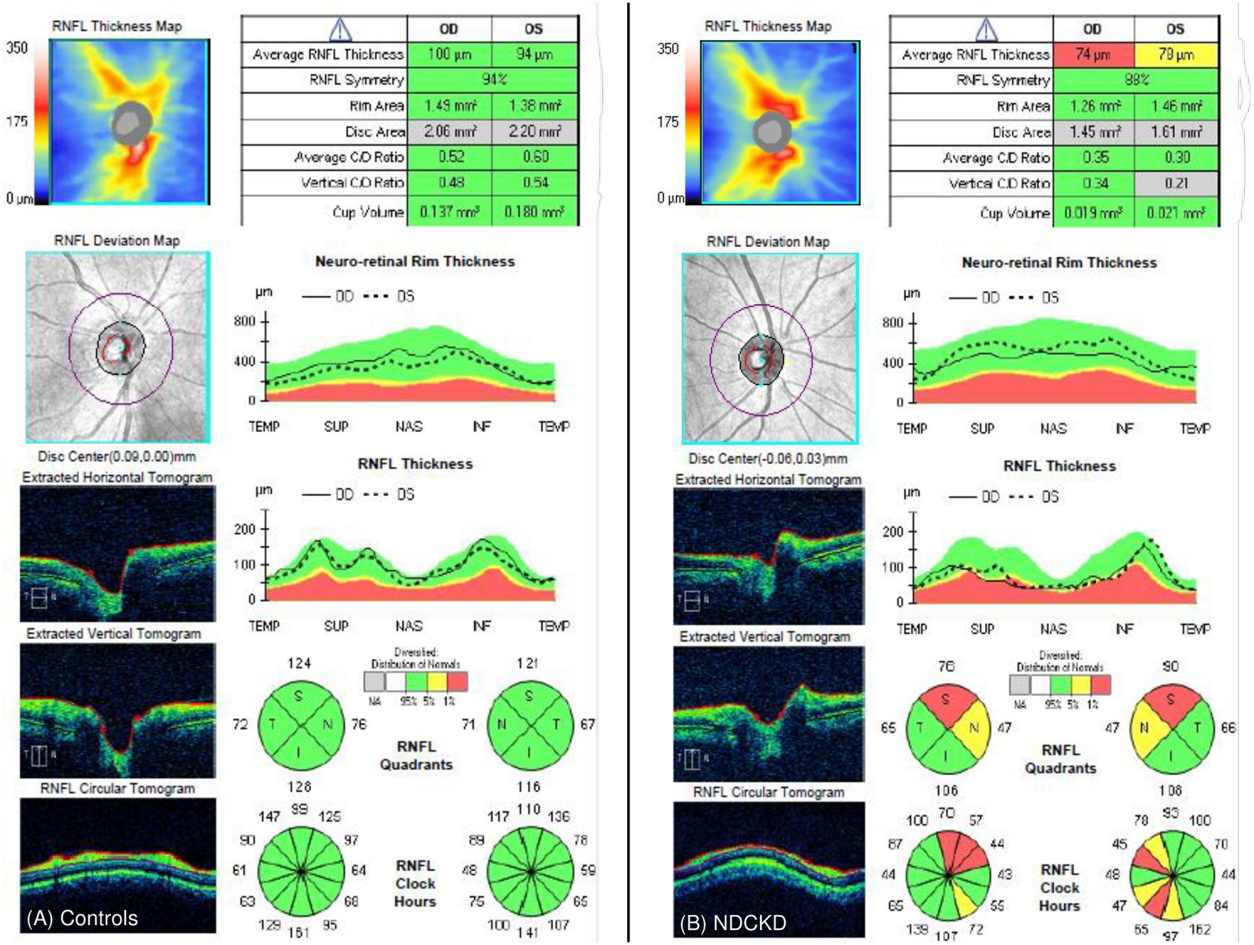
OCT image of pRNFL thickness measurement for controls and NDCKD group. (A) Control group (B) NDCKD group. Abbreviation: pRNFL = peripapillary retinal nerve fiber layer; NDCKD = non-diabetic chronic kidney disease.

**Fig 2:**
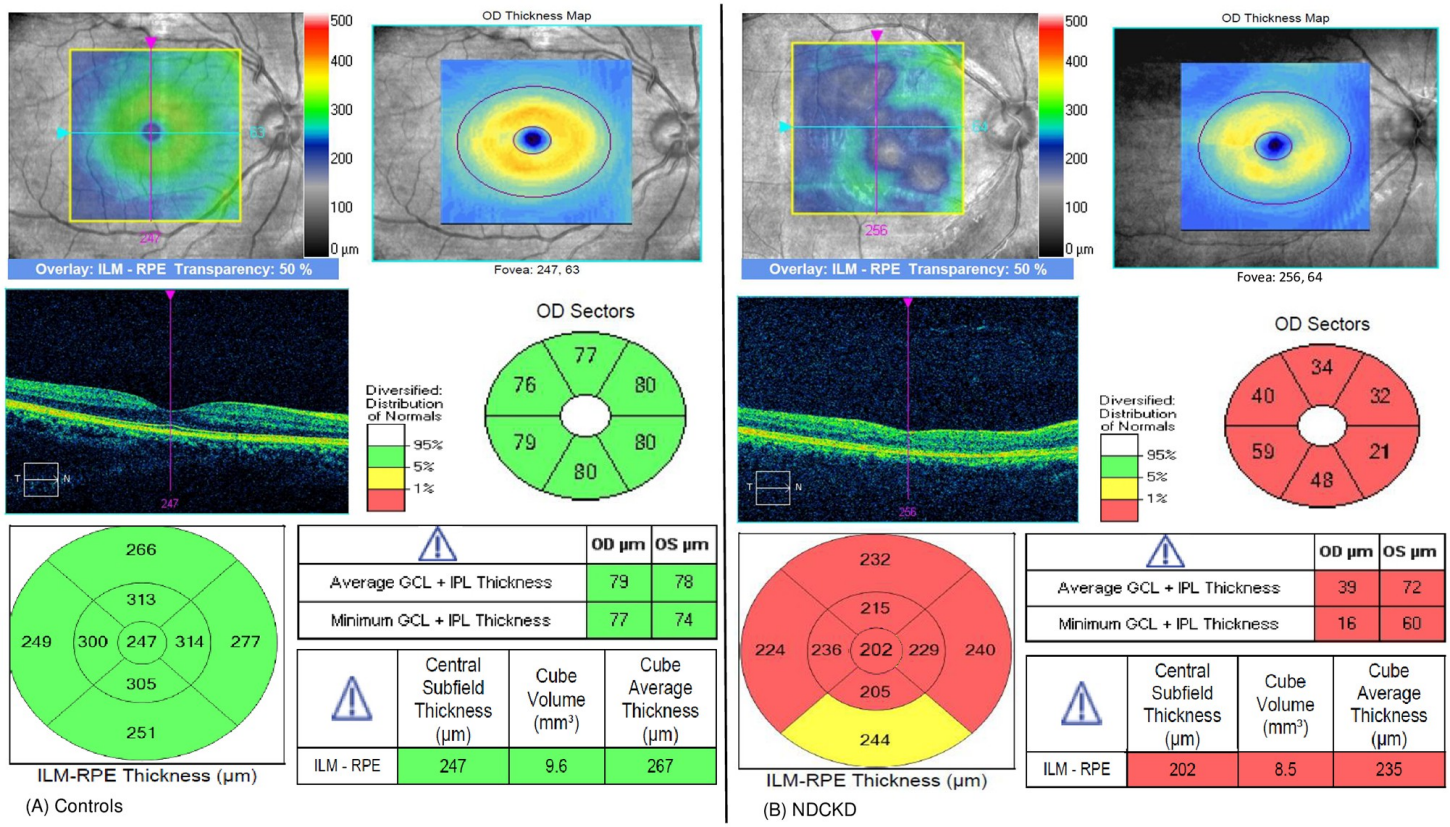
OCT image of macular thickness analysis with cross-sectional retinal layer according to position and size of the ETDRS grid for controls and NDCKD group. (A) Control group (B) NDCKD group. Abbreviation: ETDRS = early treatment diabetic retinopathy study; NDCKD = non-diabetic chronic kidney disease.

With regard to pRNFL thickness measurements, 3-dimensional (3D) cube OCT data were obtained using the “Optic Disc Cube 200 x 200 Scan” pattern in a 6 x 6mm square centred on the optic nerve head. The pRNFL thickness evaluated was the overall average pRNFL thickness and pRNFL thickness in four quadrants; superior (46°–135°), nasal (136°–225°), inferior (226°–315°) and temporal (316°–45°) [22]. MT was captured by a 6x6mm square area macular cube with 512x128 scan according to the ETDRS protocol, divided into nine subfields [23]. Central subfield thickness (CST) was defined as the average thickness of the macula in the central 1 mm ETDRS grid [24]. Average macular thickness was calculated by Cirrus OCT device based on the mean thickness of the nine subfields [25]. An automated ganglion cell analysis algorithm was used to segment and measure ganglion cell-inner plexiform layer (GC-IPL) thickness inside a 14.13 mm^2^ elliptical annulus area centered on the fovea [26]. The GC-IPL thickness, average MT and CST were taken in this study. OCT measurements were repeated three times and the most reliable result was taken. OCT images without segmentation error and a signal strength of 7 or greater were accepted. OCT image of retinal parameters were shown in Figs 1 and 2. If both eyes were eligible for the study, only the right eye was selected for statistical analysis.

### Statistical analyses

Power and sample size program was used for the calculation of sample size [27]. The sample size of 132 subjects was based on a minimum clinically relevant difference of 5μm in RNFL thickness [28] and 10μm in macular thickness [29] at a 5% level of significance and 80% power. Based on G*power analysis program [30] and partial eta-squared of 0.3 for medium effect size (basic rules of thumb) [31], a total number of 27 subjects are required to determine the difference of pRNFL thickness and MT in each stage of NDCKD patients at 80% power. Besides, 47 subjects were required to determine the correlation between pRNFL thickness and MT with the duration of CKD according to r value of 0.4 is used in sample size calculator [32]. Statistical analyses were performed using Statistical Product and Service Solutions (SPSS) software version 25.0 (IBM Corp, Armonk, NY). Visual judgment based on a histogram and shapiro-wilk test were used to verify normal distribution of the data. Categorical variables were expressed as frequency (n) and percentage (%), while numerical data was presented as mean values with standard deviation (SD). Independent sample T-test, chi-square and Fisher’s exact test were used to compare demographic data between groups while one-way analysis of variance (ANOVA), chi-square and Fisher’s exact test were performed in comparison of baseline characteristics among NDCKD stages. One-way analysis of covariance (ANCOVA) test was used to compare the differences in pRNFL and MT between NDCKD and controls, as well as between the different stages of NDCKD, with adjustment for confounders such as age, gender, blood pressure, number of antihypertensive medications, refractive error, systemic comorbidities and duration of disease. Spearman rank-order correlation coefficient was used to determine the effect of duration of NDCKD and eGFR on pRNFL thickness and MT. P values of less than 0.05 were considered statistically significant.

## Results

A total of 144 subjects were recruited but 12 subjects were excluded due to low quality image. There were 66 NDCKD patients and 66 controls. The NDCKD group had significantly more males (p = 0.015) and a significantly younger mean age (p = 0.001) than the control group. The mean number of antihypertensive medications used (p = 0.022) and others systemic diseases (p = 0.000) were significantly different between the two groups. The data was summarized in Table 1. The baseline characteristic of each stage of NDCKD was shown in Table 2. Age and duration of chronic kidney disease were found to have statistically significant different among the NDCKD stages.

**Table 1:** Demographic characteristics of subjects.

Variables	NDCKD (n = 66)	Control (n = 66)	p-value
Age ± SD (years)	45.26 ± 14.86	53.03 ± 10.22	0.001^a^*
Gender (%)			0.015^b^*
Male	41 (62.12%)	27 (40.90%)	
Female	25 (37.88%)	39 (59.10%)	
Ethnicity (%)			0.116^c^
Malay	55 (83.33%)	46 (69.70%)	
Chinese	11 (16.67%)	18 (27.28%)	
Indian	0 (0%)	1 (1.51%)	
Others	0 (0%)	1 (1.51%)	
Systolic BP ± SD (mmHg)	139.20 ± 13.93	136.14 ± 18.51	0.285^a^
Diastolic BP ± SD (mmHg)	83.86 ± 9.24	82.91 ± 9.95	0.569^a^
Number antihypertensive medications ± SD	1.71 ± 0.86	1.41 ± 0.63	0.022^a^*
BCVA ± SD (logMAR)	0.13 ± 0.15	0.12 ± 0.10	0.891^a^
Refraction, spherical equivalent (Diopter)	-0.37 ± 0.57	-0.36 ± 0.73	0.953^a^
Vertical CDR ± SD	0.41 ± 0.12	0.38 ± 0.10	0.174^a^
Other comorbidities			0.000^c^*
Dyslipidemia	25	10	
Heart disease	0	1	
Gout	4	16	
Connective tissue disease	0	10	
OCT signal strength	7.52 ± 0.85	7.71 ± 0.86	0.186^a^

*p-value < 0.05 is statistically significant.

^a^ = Independent sample T test.

^b^ = chi-square test.

^c^ = Fisher’s exact test.

n = number; NDCKD = non-diabetic chronic kidney disease; SD = standard deviation; BP = blood pressure; BCVA = best-corrected visual acuity; logMAR = Logarithm of the Minimum Angle of Resolution; CDR = cup to disc ratio.

**Table 2:** Baseline characteristic of each stage of NDCKD.

Variables	Stage 3 (n = 22)	Stage 4 (n = 22)	Stage 5 (n = 22)	p-value
Age ± SD (years)	43.91 ± 15.11	50.77 ± 15.12	41.09 ± 13.20	0.000^a^*
Gender (%)				0.057^b^
Male	13 (59.09%)	12 (54.55%)	16 (72.73%)	
Female	9 (40.91%)	10 (45.45%)	6 (27.27%)	
Ethnicity (%)				0.697^c^
Malay	18	17	20	
Chinese	4	5	2	
Indian and others	0	0	0	
Systolic BP ± SD (mmHg)	137.64 ± 14.83	137.27 ± 14.60	142.68 ± 12.19	0.453^a^
Diastolic BP ± SD (mmHg)	84.09 ± 7.9	83.41 ± 9.80	84.09 ± 10.25	0.941^a^
Number antihypertensive medications ± SD	1.64 ± 0.727	1.77 ± 0.92	1.73 ± 0.935	0.135^a^
Duration of NDCKD ± SD (year)	4.59 ± 3.26	6.06 ± 7.78	10.05 ± 6.57	0.000^a^*

*p-value < 0.05 is statistically significant.

^a^ = one-way anova.

^b^ = chi-square test.

^c^ = Fisher’s exact test.

n = number; SD = standard deviation; BP = blood pressure; NDCKD = non-diabetic chronic kidney disease.

The causes of NDCKD are summarized in Table 3. The most common etiology of NDCKD was hypertension followed by systemic lupus erythematosus, obstructive uropathy and glomerulonephritis. Other causes included gout, lgA nephropathy, polycystic kidney disease, drug-induced nephropathy, and renal carcinoma.

**Table 3:** The causes of NDCKD.

Causes	Number (percentage)
Hypertension	35 (53.03%)
Glomerulonephritis	2 (3.03%)
Obstructive	3 (4.55%)
Systemic lupus erythematosus	10 (15.15%)
Others	14 (21.21%)
Unknown	2 (3.03%)
Total	66 (100%)

The average pRNFL and all RNFL quadrants except the temporal quadrant were significantly thinner in the NDCKD group than controls (Table 4). Likewise, CST, average MT and GCL-IPL thickness were significantly thinner in the NDCKD group than the control group. The value of pRNFL thickness appear to have higher variability for all the quadrants in the NDCKD group in comparison to the control group as there have broader range of eGFR value, different stages and wider range of age groups among NDCKD subjects.

**Table 4:** Comparison of mean pRNFL, CST, MT and GC-IPL thickness measurement between NDCKD and control groups.

Parameters, μm (mean ± SD)	Quadrants	NDCKD (n = 66)	Control (n = 66)	p-value
pRNFL thickness	Superior	110.74 ± 23.35	117.36 ± 16.17	0.022*
	Temporal	66.70 ± 13.63	68.79 ± 12.00	0.452
	Nasal	65.97 ± 12.90	69.35 ± 10.17	0.006*
	Inferior	117.44 ± 25.98	126.15 ± 14.75	0.006*
	Average	90.36 ± 14.93	95.42 ± 9.87	0.005*
CST		231.89 ± 26.72	243.30 ± 21.05	0.006*
Average MT		268.88 ± 20.21	274.92 ± 12.79	0.020*
Average GC-IPL thickness		75.48 ± 12.44	81.56 ± 6.48	0.001*

One-way ANCOVA adjusted for age, gender, blood pressure, number of antihypertensive medications, refractive error and other systemic comordities.

*p-value < 0.05 is statistically significant.

SD = standard deviation; n = number; NDCKD = non-diabetic chronic kidney disease; pRNFL = peripapillary retinal nerve fiber layer; MT = macular thickness; CST = central subfield thickness; ANCOVA = analysis of covariance; GC-IPL = ganglion cell-inner plexiform layer.

Within the NDCKD group, each group (stage 3, stage 4 and stage 5) had 22 patients. Average pRNFL thickness, average MT, average GC-IPL thickness and all pRNFL quadrants except temporal differed significantly among CKD stages (Table 5). Bonferroni correction revealed significant differences in superior quadrant pRNFL thickness, nasal quadrant pRNFL thickness, average pRNFL thickness, average MT and average GC-IPL thickness between CKD stage 3 and 5 while similar results between CKD stage 4 and 5 except nasal quadrant of pRNFL is not significantly different (Table 6).

**Table 5:** Comparison of mean of pRNFL, CST, MT and GC-IPL thickness among stage 3, stage 4 and stage 5 NDCKD.

Parameters, μm (mean ± SD)	Quadrants	NDCKD			p-value
		Stage 3 (n = 22)	Stage 4 (n = 22)	Stage 5 (n = 22)	
pRNFL thickness	Superior	118.05 ± 15.64	114.27 ± 28.39	99.91 ± 21.11	0.007*
	Temporal	69.68 ± 11.54	65.77 ± 12.99	64.64 ± 16.08	0.340
	Nasal	70.23 ± 13.74	64.64 ± 12.41	63.05 ± 11.93	0.030*
	Inferior	120.55 ± 25.00	118.91 ± 27.53	112.86 ± 25.92	0.047*
	Average	95.05 ± 11.60	90.91 ± 16.76	85.14 ± 14.92	0.006*
CST		234.05 ± 22.52	239.45 ± 22.81	222.18 ± 31.93	0.091
Average MT		275.15 ± 16.41	273.59 ± 14.00	257.91 ± 24.67	0.001*
**Average GC-IPL thickness**		80.09 ± 7.62	77.95 ± 8.71	68.41 ± 16.25	0.001*

One-way ANCOVA adjusted for age, gender, blood pressure, number of antihypertensive medications, duration of CKD, refractive error and systemic comorbidities.

* p-value < 0.05 is statistically significant.

SD = standard deviation; NDCKD = non-diabetic chronic kidney disease; pRNFL = peripapillary retinal nerve fiber layer; MT = macular thickness; CST = central subfield thickness; ANCOVA = analysis of covariance; GC-IPL = ganglion cell-inner plexiform layer.

**Table 6:** Post hoc Bonferroni test for differences in pRNFL, CST, MT and GC-IPL thickness between NDCKD stages.

Parameters	Quadrants	Post hoc Bonferroni Test		
		p1	p2	p3
pRNFL thickness	Superior	0.905	0.006*	0.004*
	Temporal	0.394	0.504	0.149
	Nasal	0.154	0.171	0.009*
	Inferior	0.730	0.043	0.019
	Average	0.424	0.016*	0.002*
CST		0.563	0.033	0.098
Average MT		0.760	0.002*	0.001*
Average GCL + IPL thickness		0.391	0.004*	0.000*

p1 = Comparison of stage 3 and stage 4.

p2 = comparison of stage 4 and stage 5.

p3 = comparison of stage 3 and stage 5.

*Bonferroni adjusted p-value < 0.017 is statistically significant.

pRNFL = peripapillary retinal nerve fiber layer; CST = central subfield thickness; MT = macular thickness; GC-IPL = ganglion cell-inner plexiform layer.

**Fig 3:**
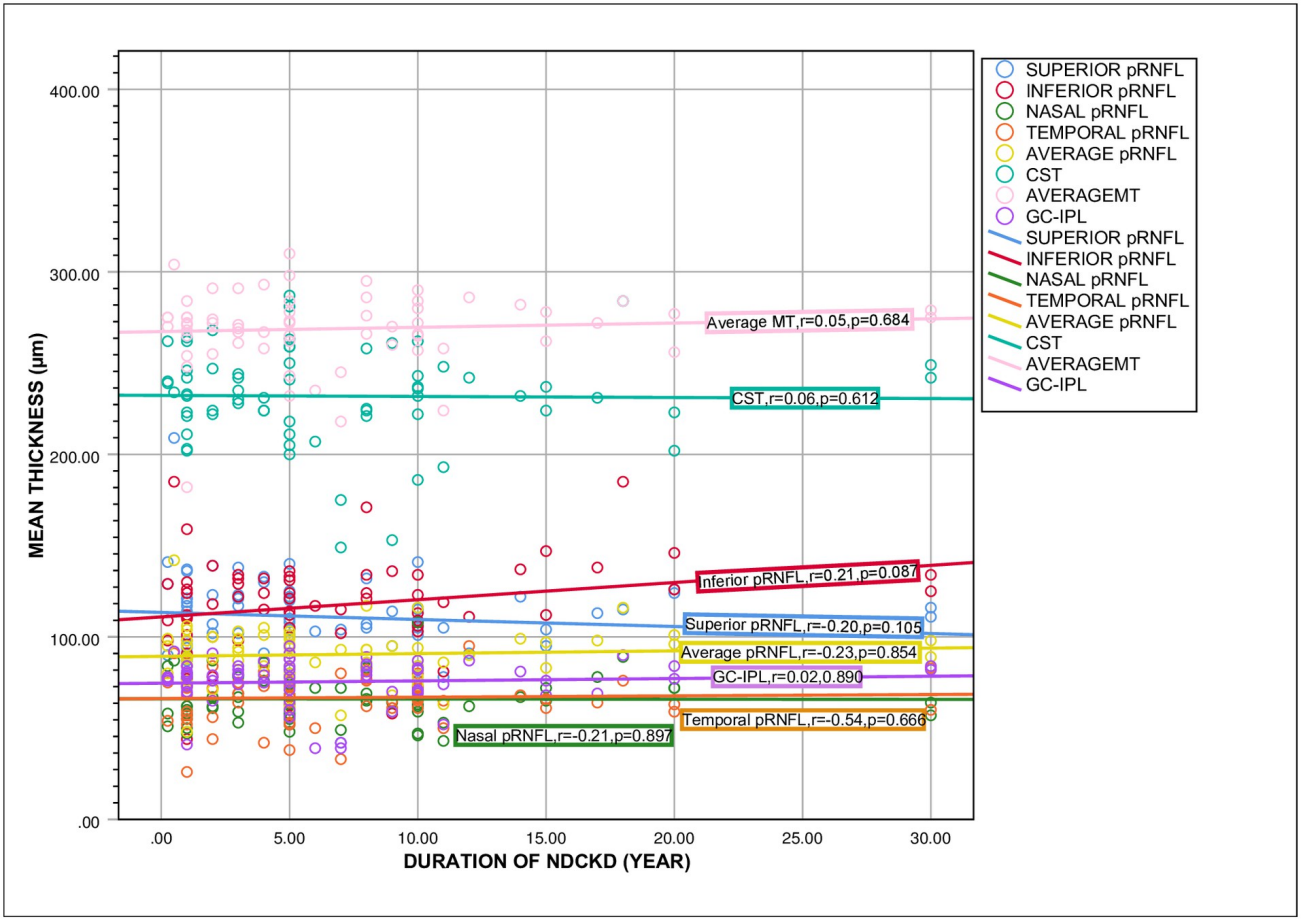
The relationship between pRNFL, CST, MT and GC-IPL thickness with the duration of CKD. The p-value was calculated by using spearman rank-order correlation coefficient. * p < 0.05 is statistically significant. Abbreviation: pRNFL = peripapillary retinal nerve fiber layer; MT = macular thickness; CST = central subfield thickness; NDCKD = non-diabetic chronic kidney disease; GC-IPL = ganglion cell-inner plexiform layer; r = spearman correlation coefficient.

**Fig 4:**
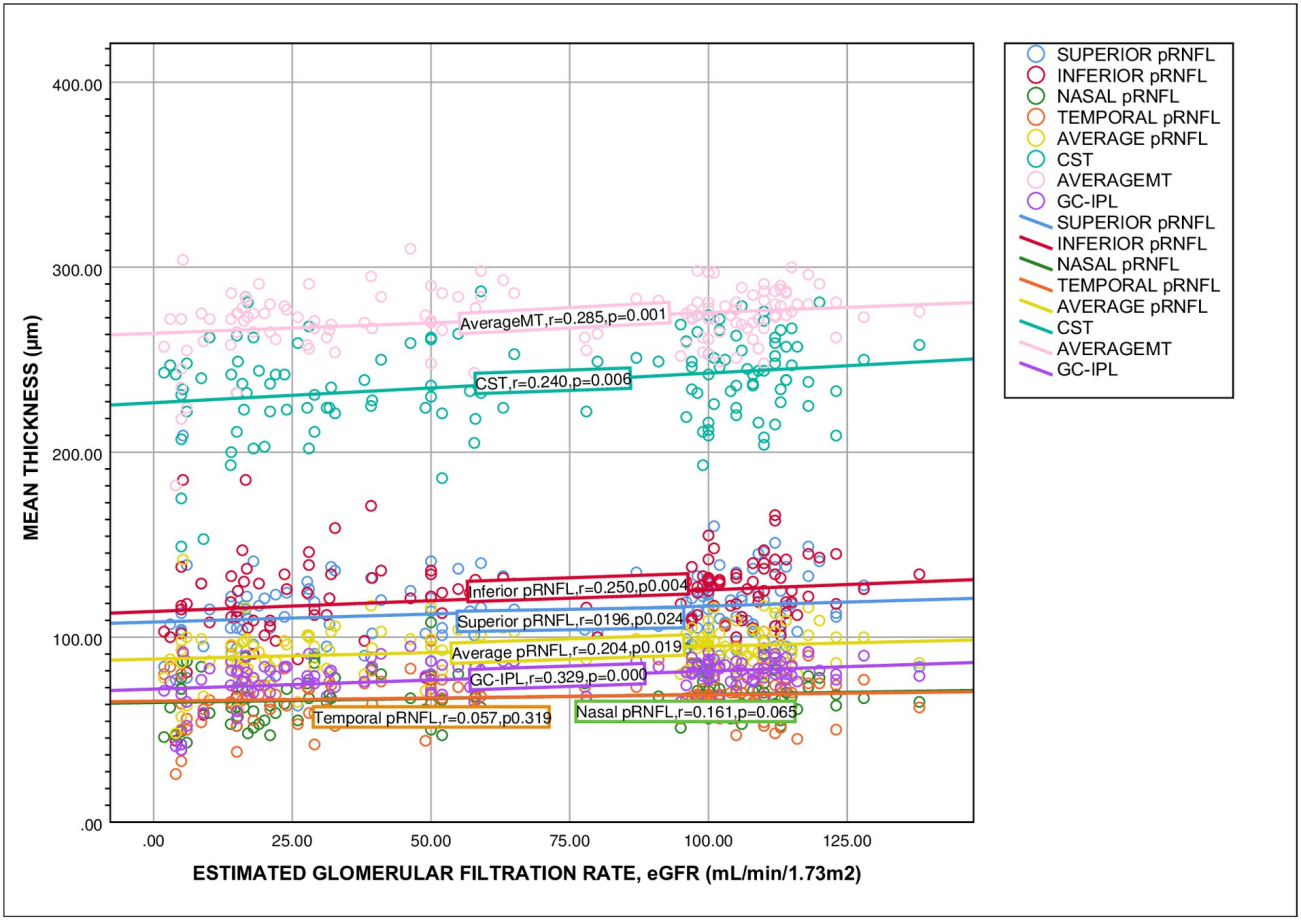
The relationship between pRNFL, CST, MT and GC-IPL thickness with the eGFR. The p-value was calculated by using spearman rank-order correlation coefficient. * p < 0.05 is statistically significant. Abbreviation: pRNFL = peripapillary retinal nerve fiber layer; MT = macular thickness; CST = central subfield thickness; GC-IPL = ganglion cell-inner plexiform layer; r = spearman correlation coefficient.

We observed no statistically significant correlation between pRNFL thickness, CST, MT and GC-IPL with the duration of NDCKD (Fig 3). In a subanalysis, we found that superior, inferior, average pRNFL, CST, average MT and GC-IPL have significant positive correlation with eGFR (Fig 4).

## Discussion

CKD has been linked with specific optic neuropathies such as uremic optic neuropathy, ischemic optic neuropathy and glaucoma, as well as macular thinning [33–35]. pRNFL and macular thickness parameters may act as early biomarkers for risk stratification in CKD. To the best of our knowledge, this is the first study to demonstrate the differences in pRNFL thickness and MT between patients with different stages of NDCKD as well as the correlation between pRNFL thickness and MT with the duration of NDCKD.

We observed that the pRNFL thickness was significantly thinner in the NDCKD group than the control group in all quadrants except the temporal quadrant. This is in keeping with the literature. Demir et al. noted a statistically significant thinning in all quadrants and average RNFL thickness among non-diabetic patients with ESRF on regular HD [11] while Atilgan et al. reported a statistically significant thinning in the inferior, temporal, and average RNFL values among a similar group [36]. The superior, nasal, and inferior quadrants and average RNFL thickness were greater in the non-diabetic non-CKD group than the chronic renal failure group as stated by Gadelha et al [37]. These studies were all conducted among patients with end stage renal failure. Our study adds to the literature by demonstrating that even prior to the onset of dependence on dialysis, the pRNFL is thinner in patients with CKD than controls. Postulated reasons for a thinner pRNFL in these patients include subclinical uremic optic neuropathy and chronic vascular insufficiency related to CKD-induced complications such as anemia, hypertension, or atherosclerosis [12, 38].

In general, we observed that the NDCKD group had a thinner CST, average MT and average GC-IPL thickness than controls. A 10% reduction in retinal thickness and macular volume has been found in all stages of NDCKD [39]. In contrast, Pahor et al. observed fovea-sparing reduction in retinal thickness among ESRF patients [40]. Thinning of the macula region including CST among those with NDCKD may be due to subclinical ischemic retinopathy [36]. Microvascular injury and choroidal thinning in CKD patients may compromise the blood supply to the retinal neural tissue resulting in retinal atrophy [39, 41, 42]. This is supported by observations of decreased retinal vessel density, parafoveal retinal thickness and macular ganglion cell-inner plexiform layer thickness among patients with CKD [43–45].

We found significant differences in average pRNFL thickness, average MT, average GC-IPL thickness and all pRNFL quadrants except temporal between different stages of CKD. As expected, those with end stage renal failure had the thinnest PRNFL and macular thickness. In our subanalysis, pRNFL thickenss of superior quadrant, inferior quadrant, average pRNFL, CST, average macular thickness and GC-IPL showed significant positive correlation with eGFR. Renal dysfunction has also been correlated with decreased choroidal thickness [39] and pRNFL thickness [46–48]. This may partly be attributed to the sympathetic nervous system overactivation which is characteristic of CKD [49]. As the outer retinal layers are supplied by choriocapillaries, changes in choriocapillary function may indirectly affect the retinal thickness. While Paterson et al. showed that retinal microvascular changes in advanced CKD (stages 4 & 5) causing inner retinal thinning [50]. We found that there were no statistically significant correlations between pRNFL thickness and MT and the duration of NDCKD. This is in keeping with the results of Pahor et al. [40]. Atilgan et al. reported that pRNFL thickness and MT were not significantly related to the duration of HD treatment except in the superior pRNFL quadrant [36]. Therefore, the thinner retinal parameter in OCT might indicate more advanced stage of NDCKD rather than the disease duration.

The strengths of our study are its objective and reliable assessment of pRNFL thickness with validated instruments and its use of statistical tests that adjust for the effect of confounders such as age, gender [51], blood pressure and number of antihypertensive medications [52], refractive error and systemic comorbidities [53]. However, the pRNFL is affected by multiple factors, so although we excluded major causes of retinopathy such as diabetes [54, 55], adjustment for all potential factors affecting the pRNFL may be impossible. Additionally, hemodialysis has been associated with pRNFL and macular thinning [36, 56], possibly due to its effect on retrobulbar recirculation [57]. However, the literature on this is controversial, with other studies reporting no effect on pRNFL [58, 59] and central retinal thickness [60]. As all patients with end stage renal disease were on hemodialysis, the effect of the latter may have an additive effect on pRNFL and MT thinning. The different demographic data between NDCKD and control groups are still one of the limitations of this study although they were adjusted for statistical analysis. Finally, the cross-sectional nature of our study limits inferences of a temporal nature. Longitudinal studies may provide greater information about the RNFL changes which occur in CKD.

OCT monitoring of pRNFL and macular thickness may provide non-invasive risk stratification of patients with CKD prior to visual compromise, thus allowing disease optimization prior to onset of irreversible blinding complications. In addition, the thinner pRNFL observed in NDCKD patients may complicate the interpretation of structural changes in CKD patients undergoing glaucoma evaluation.

## Conclusion

CST, average MT, average GC-IPL thickness, average pRNFL thickness, and all pRNFL quadrants except the temporal were significantly thinner in NDCKD patients than controls. These parameter changes were associated with the severity but not the duration of CKD.

## Supporting information

S1 Table Minimal data set. (XLSX)
